# Upregulation of E‐cadherin in bronchoalveolar lavage fluid‐derived exosomes in patients with lung cancer

**DOI:** 10.1111/1759-7714.13220

**Published:** 2019-11-06

**Authors:** Ying Zhang, Ziyu Liu, Shanyu Li, Manning Wang, Dayou Dai, Hongyu Jing, Lingyun Liu

**Affiliations:** ^1^ Key Laboratory for Molecular Enzymology and Engineering, The Ministry of Education, School of Life Science Jilin University Changchun China; ^2^ The First Hospital of Jilin University, Department of Pediatrics Jilin University Changchun China; ^3^ Department of Clinical Medicine Southern Medical University Guangzhou China; ^4^ The First Hospital of Jilin University, Department of Respiratory Medicine Jilin University Changchun China; ^5^ The First Hospital of Jilin University, Department of Andrology Jilin University Changchun China

**Keywords:** BALF, E‐cadherin, exosome

## Abstract

**Background:**

Lung cancer features extremely high rates of morbidity and mortality. Bronchoalveolar lavage fluid (BALF), obtained by bronchoscopy and bronchoalveolar perfusion, can provide information on the cellular components of the lung microenvironment to assist with diagnosis and treatment of lung cancer.

**Methods:**

BALF was performed using a flexible bronchofiberscope. Exosomes were collected by ultracentrifugation. ELISA detected the amount of E‐cadherin. Transmission electron microscopic, ELISA and WB were conducted to identify the existence of the exosomes. Transwell and Wound healing assays were used to detect the ability of migration and invasion.

**Results:**

We identified the existence of exosomes in BALF. Furthermore, we observed larger amounts of E‐cadherin in the BALF obtained from patients with lung cancer than in the control obtained from the healthy side of pneumonia. Exosomes from lung cancer groups promoted the migration and invasion of A549 cancer cells.

**Conclusion:**

The exosomes from lung cancer BALF promoted the migration and invasion of A549 cancer cells by carrying E‐cadherin. E‐cadherin on the surface of exosomes may act through a VE‐cadherin dependent mechanism and induce lung cancer metastasis.

## Introduction

Lung cancers are associated with extremely high morbidity and mortality.^1^ In China, the diagnosis of lung cancer usually occurs very late in the course of the disease, by which time around two‐thirds of patients are no longer candidates for radical surgery.^2^ Lung computed tomography (CT) testing has facilitated early detection of lung cancer. Bronchoscopy, mediastinoscopy, or thoracentesis biopsy are the most reliable diagnostic methods. However, these are highly specialized procedures with each carrying different risks. They cannot be used to screen or treat lung cancer, or for continuous disease monitoring.^3^ Validation of early and noninvasive means of diagnosing and pathologically classifying lung cancers may help improve long‐term survival and quality of life.

Fiberoptic bronchoscopy is a fairly recent lung examination technique that allows the direct observation of airway tissue morphology and lung tumor biopsy samples can be obtained when used in combination with ultrasound and biopsy forceps.

Chemotherapy has great potential for precise treatment of lung cancer.^4^ The bronchoalveolar lavage fluid (BALF) obtained during fiberoptic bronchoscopy reflects the microenvironment of the respiratory system and carries alveolar surface liquid, along with its various cellular components. BALF is a source of inflammatory cells (alveolar macrophages, neutrophils, and monocytes) as well as other soluble components within the alveoli.^5^ Inflammatory cells obtained from BALF can be used to assess acute lung injury, the severity of rejection after lung transplantation, soluble components such as proteins and cytokines, and can be used to predict and diagnose bronchial asthma. For lung cancer staging, pathogenic microorganisms such as bacteria, tuberculosis, and parasites, isolated from BALF, can be used for rapid diagnosis. Proteomics and flow cytometry have been used in some studies to analyze the various cells and cellular components in BALF obtained from patients with lung cancer.

Exosomes are present in all body fluids, and carry proteins, lipids, and RNA. Exomes also act as mediators of intercellular communication, affecting both normal and pathological conditions. Some scholars have successfully isolated exosomes in BALF from patients with silicosis and idiopathic pulmonary fibrosis; however, the role of exosomes in BALF obtained from patients with lung cancer is unclear, as is the role of exomes in lung cancer metastasis.

Exosomes obtained from patients with cervical cancer carry soluble E‐cadherin and promote metastasis.^6^ In our study, we isolated exosomes from BALF obtained from patients with lung cancer in order to isolate E‐cadherin which could potentially be used for lung cancer typing. In addition, we explored the exosome promoting lung cancer metastasis to enable the development of future therapy targets for individualized lung cancer treatments.

## Methods

### Patients

We collected BALF samples from patients with lung cancer from November 2018 to December 2018. The Human and Animal Ethics Review Committee of the First Hospital of Jilin University approved this study. All BALF samples were collected during diagnostic bronchoscopy procedures.

### Bronchoalveolar lavage fluid (BALF)

BALF was performed using a flexible bronchofiberscope after administration of local anesthesia with lidocaine. We instilled 50 mL fractions of sterile saline into the right middle lobe or the left lung segment. BALF was retrieved using gentle syringe suctioning and transferred into sterile containers prior to storage at 4°C.

### Exosome isolation

The BALF was centrifuged using a Beckman Coulter Avanti‐J‐26XPI centrifuge at 3000 rpm at 4°C for five minutes to remove detached cells. The supernatant was recovered and sequentially centrifuged as follows: 500 rcf/10 minutes, 12 000 rcf/20 minutes and 100 000 rcf/90 minutes (× 2).

### ELISA

BALF and exosomes solution were added to a Human E‐Cadherin SimpleStep ELISA Kit (ab233611), according to the manufacturer's instructions. The concentrations of E‐cadherin from two types of BALF were measured in duplicate and interpolated from the E‐cadherin standard curves and corrected for sample dilution. The interpolated dilution factor corrected values were then plotted (mean ± SD, *n* = 3).

### Transmission electron microscopy

Using a 100 mesh sample‐loaded copper mesh, 20 μL of the freshly obtained exosome solution was diluted with the same volume of PBS, dropped onto the copper mesh, left at room temperature for one minute, and then air‐dried at room temperature. Then, we used a dropper to take 20 μL of 3% (w/v) sodium phosphotungstate solution and this solution was dropped onto the copper grid for one minute and excess liquid gently absorbed using filter paper. After we placed the copper mesh under a transmission electron microscope for evacuation, we observed and photographed the morphology of the exosomes.

### Protein concentration determination

Exosomes were lysed by repeated freezing and thawing in liquid nitrogen on at least three occasions and the lysate then centrifuged at 13 000 rpm using Thermo Scientific Sorvall Legend Micro17/21. The supernatant was added to a BCA protein Assay Kit (P0012), according to the manufacturer's instructions.

### Western blot (WB)

Exosomes were lysed with buffer containing 260 mM Tris–HCl, pH 6.8, 0.8% SDS (w/v), and 40% glycerol, supplemented with protease inhibitors: 1 μg/mL aprotinin, 1 μg/mL leupeptin, 1 μg/mL pepstatin, and 1 mM phenylmethyl sulfonyl. Equal amounts of protein (30 μg) were resolved by SDS‐PAGE for blotting using anti‐CD63 (1:200, santa), CD81 (1:1000, santa), TSG101 (1:1000, proteintech), and E‐cadherin (1:1000, proteintech). HRP‐conjugated anti‐rabbit IgG antibody were used as secondary antibodies (1:5000, proteintech).

### Migration assay

A total of 500 000 cells were seeded per well in 24 well plates in triplicate. After 24 hours, the medium was replaced by medium without FBS and maintained overnight. A wound was then made in the monolayer with a pipette tip, and the medium was replaced with serum‐free media and exosomes from each group. Pictures of the wounds were taken at 0 and 24 hours using an Olympus IMT‐2 microscope. Wound closure was measured using ImageJ software. Results represent the migrated distance between 0 and 24 hours, expressed as a percentage relative to A549.

### Invasion assay

A total of 8 μm pore inserts were covered with 50 μL Matrigel (356 231, Corning) at a final concentration of 1.25 mg/mL. Matrigel was incubated for one hour at 37°C. Once the Matrigel was polymerized over the Matrigel layer, 30 000 cells with exosomes from each group were seeded in 100 μL medium without FBS. Complete medium (supplemented with FBS, which stimulates the cells to cross the Matrigel layer) was added to the well under the insert, covering the bottom of the insert. Twenty‐four hours after seeding, cells were fixed with absolute ethanol. The top of the insert was cleaned with a cotton bud to remove the Matrigel and any cells that did not cross the layer. Images were taken of the entire bottom of the insert where the invading cells were located, using an Olympus IMT‐2 microscope (amplification 20X) and X80 software.

### Statistical analysis

Data is presented as mean value ± standard deviation (mean ± SD) in this study. GraphPad Prism 7.0 (La Jolla, CA, USA) was recruited to conduct statistical analysis. The differences between groups (≤2) were analyzed using a Student's *t*‐test, while a one‐way ANOVA was used for three or more (≥3) groups. A *P*‐value less than 0.05 was considered to be statistically significant.

## Results

### Presence of E‐cadherin in BALF

**Figure 1:**
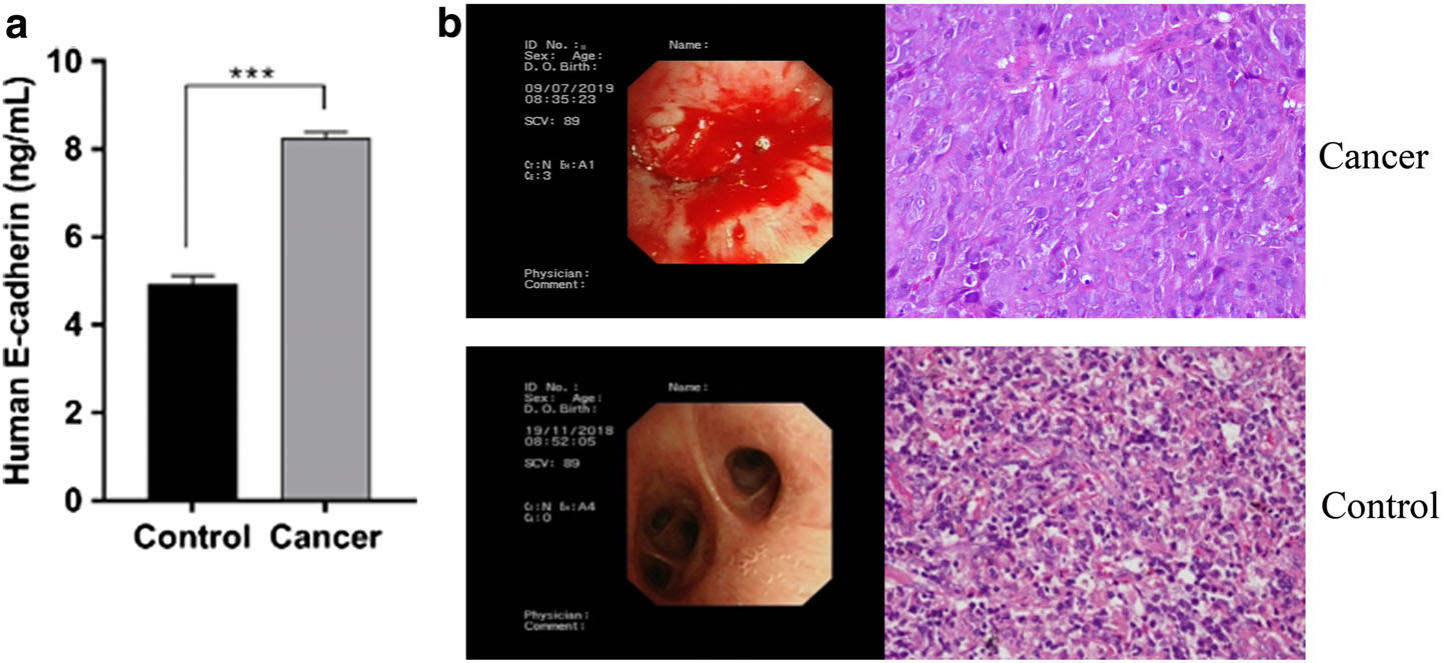
E‐cadherin increased in BALF from the lung cancer patients in contrast to the controls. (**a**) The concentrations of E‐cadherin from two types of BALF were measured in duplicate, interpolated from the E‐cadherin standard curves and corrected for sample dilution. (**b**) Representative images of endoscopic view and HE views (magnification 40x)

In patients with lung cancer, E‐cadherin may facilitate metastasis. To verify the presence of E‐cadherin in BALF, we used the ELISA technique. As Figure 1a shows, there was a significant upregulation in the concentration of E‐cadherin in the same amount of BALF obtained from patients with lung cancer and control subjects, consisting of patients with the healthy side of pneumonia. Imaging of abnormal soft tissue and postoperative pathology showed that patients with lung cancer had significantly higher concentrations of E‐cadherin in their BALF than did the control subjects. We further identified this on the views of the endoscopic and HE stained images and found the tissue obtained by bronchoscopy from cancer groups have typical pathological features as Figure 1b.

### Isolation and characterization of exosomes from BALF

**Figure 2:**
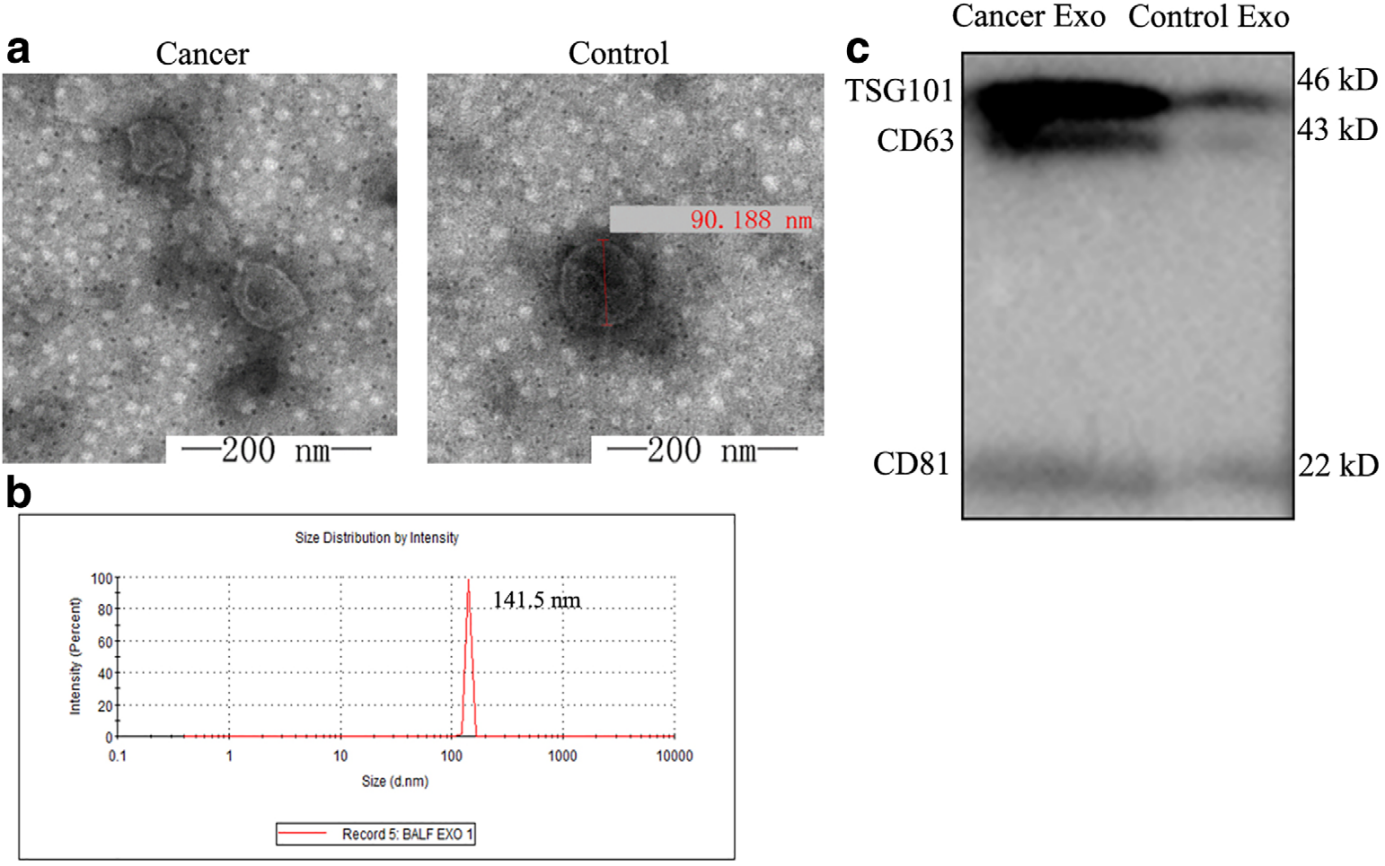
Exosomes were successfully isolated and characterized from BALF. (**a**) Transmission electron microscopic view of exosomes in both types of samples at a magnification of 120 000x. (**b**) The size distribution of isolated exosomes using Zetasizer Nano ZS90. (**c**) Total exosomes isolated from equal amounts of BALF were analyzed by Western blot for the presence of the exosomal markers CD63, CD81 and TSG101.

Exosomes can carry and transport E‐cadherin, thereby promoting cancer metastasis. To verify whether the increased concentration of E‐cadherin in BALF obtained from patients with lung cancer was carried and transported by exosomes, we examined exosomes in BALF by transmission electron microscopy (Fig 2a), and observed cup‐shaped vesicles with diameters of 20–100 nm and two‐layer membranes.

To further characterize the size of the vesicles, as depicted in Figure 2b, we used a zeta potential particle size analyzer. Specifically, we measured the Brownian motion of the particles within the BALF sample using dynamic light scattering techniques. Indirect fitting yielded the particle sizes and distributions. As Figure 2b shows, the particles were uniformly dispersed, distributed mainly at 130–150 nm, with a single‐peak diameter and a peak of 141.5 nm. This represented the hydrated vesicles, covalently bonded to the surrounding water molecules. To further verify whether the vesicle structure isolated was exosomes, the presence of exogenous marker proteins CD63, CD81, and TSG101 were detected by WB (Fig 2c). The isolated vesicles exhibited distinct bands at 43 kDa, 22 kDa, and 46 kDa.

### Exosomes carrying E‐cadherin increase the amount of total E‐cadherin in BALF

**Figure 3:**
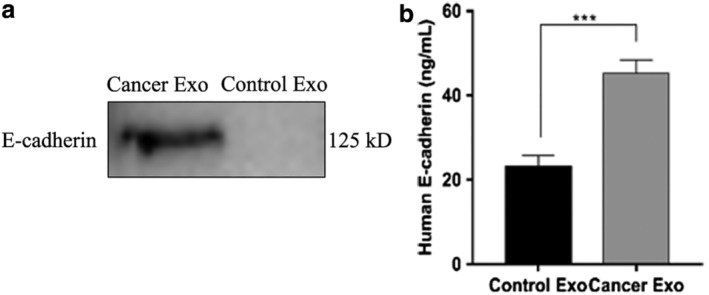
The concentration of E‐cadherin significantly increased in lung cancer BALF. (**a**) Western blot analysis of E‐cadherin in exosomes isolated from equal amounts of BALF. (**b**) The concentrations of E‐cadherin from exosomes isolated from two types of BALF were measured in duplicate.

We measured the protein concentrations on exosomes obtained from patients with lung cancer and controls to test whether exosomes obtained from patients with lung cancer carried more E‐cadherin. As Figure 3a,b shows, patients with lung cancer exhibited significantly higher concentrations of E‐cadherin on exosomes, compared to the control group by WB and ELISA. Thus, increased E‐cadherin content in BALF obtained from patients with lung cancer was attributable to increased exocrine secretion and increased E‐cadherin on the exosome surfaces.

### Exosomes carrying E‐cadherin promote the migration and invasion of A549 cancer cells

**Figure 4:**
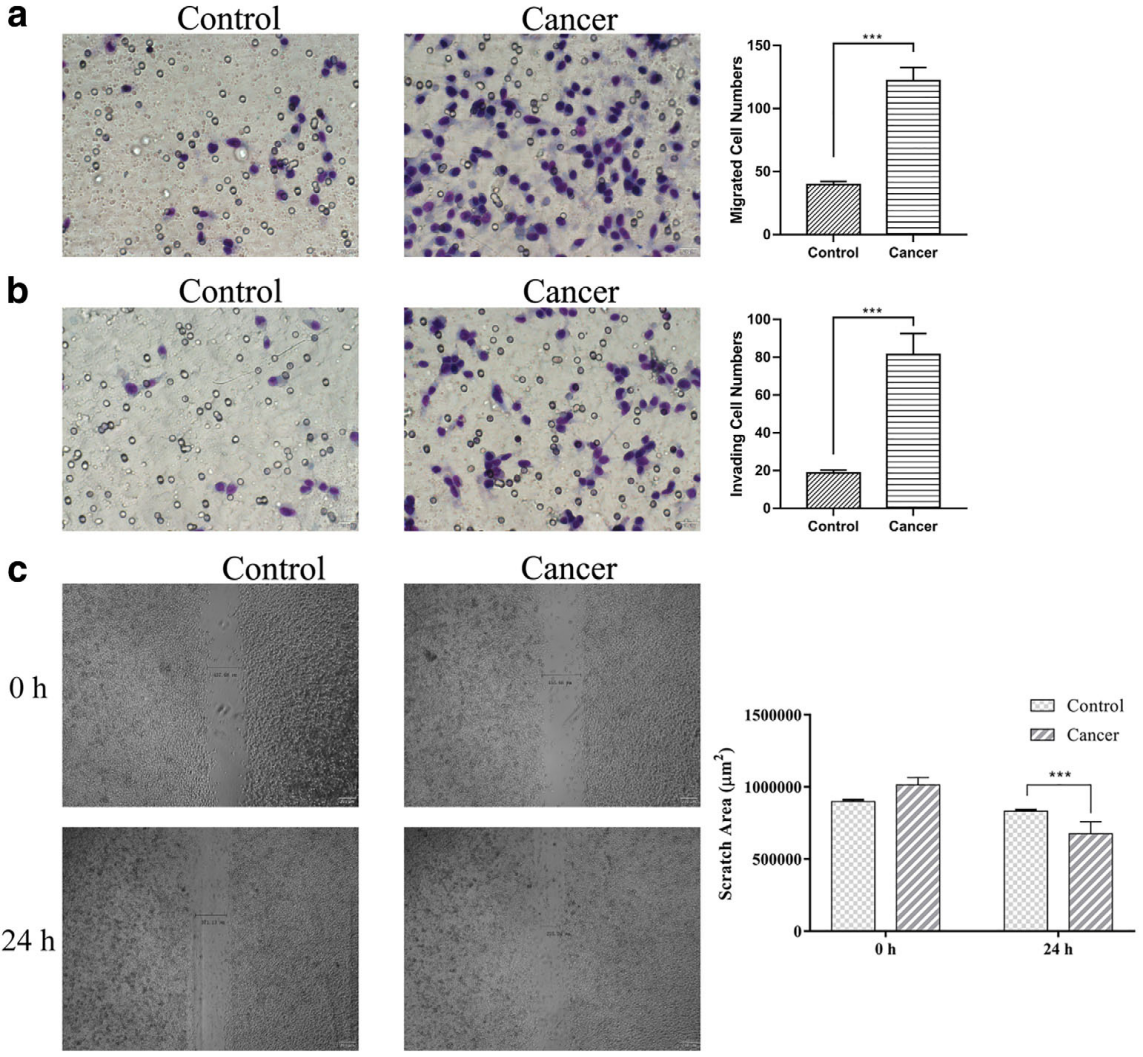
The exosomes from lung cancer BALF promoted the metastasis of A549. (**a**) The representative images of migrated cells treated by exosomes isolated from equal amounts of BALF by Transwell assay without Matrigel matrix are shown. (**b**) The invasion assays were conducted by exosomes isolated from equal amounts of BALF by Transwell assay with Matrigel matrix. (**c**) The distance of wound healing assay was measured cocultured with exosomes from each group. Significant differences were determined using an unpaired *t*‐test with Welch's correction. Asterisks indicate significant differences where *P*‐values are <0.05 (*) and <0.01 (**). (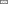) Control and (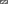) cancer.

To confirm the effect of exosomes carrying E‐cadherin, we tested the migratory and invasive capacity of the lung cancer cell line A549 with exosomes isolated from the different groups. Furthermore, the migration ability of the exosomes could be achieved by the invasion assay without matrigel matrix. The cells which had migrated could be counted after 24 hours of seeding as shown at the top of the insert (Fig 4a). At the same time, A549 cells treated in each group were embedded in a Matrigel/collagen matrix and the cell expansion counted. Comparison of the total number of migrating and invading cells revealed that cells from cancer patients mixed with exosomes carried E‐cadherin and were significantly more effective at invasion than the cells mixed with exosomes from healthy patients (Fig 4b). A wound healing assay was used to measure cell migration, which is essential for many biological processes including tumor invasion and metastasis. The group treated by exosomes from cancer patients had strong migratory capacity compared to the control group. To confirm the migration ability affected by exosomes from each goup, we performed a Scratch assay to measure the cells treated with exosomes from each group. The analyzed distances treated by exosomes from cancer patients were longer than the control group (Fig 4c). The assays described above illustrate that the exosomes from lung cancer promoted the metastasis of the A549 cancer cells.

## Discussion

The technique for obtaining exosomes from BALF in patients and animals with various inflammatory diseases and lung cancers has been widely reported in the literature. However, there are no reports on the successful isolation of exosomes from BALF obtained from patients with lung cancer. In this study, high concentrations of pure exosomes were successfully isolated from BALF obtained by fiberoptic bronchoscopy and alveolar lavage.

Exosomes are associated with progression of airway mucosal diseases, such as asthma and chronic obstructive pulmonary disease.^7^, ^8^, ^9^, ^10^ Furthermore, some reports have compared two different types of sample using differential proteomics. Changes in the proteins carried by exosomes associated with small cell lung cancer cell lines have revealed a marker protein that may be used to differentiate several non‐small cell lung cancers.^11^ This has provided a new assay for the diagnosis and pathological classification of lung cancer. Compared with lung biopsy, the incidence of lung cancer dissemination and metastasis may be lowered through ongoing examination of BALF; additionally, this technique has good diagnostic utility for lesions distant from the main bronchus.

In patients with lung cancer, exocrine concentrations are significantly increased compared to normal tissues. Moreover, the genetic information carried by exosomes promotes tumor formation, metastasis and invasion. This can promote angiogenesis at the tumor site and epithelial‐mesenchymal transition (EMT) in the microenvironment of the tumor. At the same time, exosomes also affect the cellular immune response by carrying tumor antigens. Tumor antigens affect cellular immune responses. As mediators of intercellular communication, exosomes play a critical role in establishing the tumor microenvironment.^12^ in vitro studies have confirmed that exosomes obtained from lung cancer cells may activate NF‐κB‐TLR, JAK‐STAT, Fas/FasL, PI3K/AKT/GSK3β,^13^ and other signaling pathways.^14^, ^15^ We confirmed, through examination of clinical samples, that exosomes obtained from patients with lung cancer carry E‐cadherin. This increases the E‐cadherin concentration within the tumor microenvironment, setting the stage for lung cancer metastasis. It is worth giving thought to whether we can alter the extracellular microenvironment by designing targeted drugs against exosomes, thereby blocking intercellular communication and providing an effective strategy for inhibiting the metastasis of lung cancer. Furthermore, in our study, we identified that the exosomes carried more E‐cadherin and could promote the capacity of migration and invasion in lung cancer cell lines.

We compared the concentrations and contents of exosomes isolated from patients with lung cancer and pneumonia healthy side lung tissue, and then correlated this information with the clinical pathological findings. BALF obtained from patients with lung cancer contained significantly more exosomes than BALF obtained from patients with pneumonia healthy side lung tissue. Furthermore, interesting discoveries have been made in the field of signal transporting. Several groups have demonstrated that exosomes upregulate prometastatic and tumor angiogenesis pathways,^16^ even transforming normal cells into cancer cells.^17^, ^18^ Our complementation assay confirmed exosome testing may be able to differentiate various lung cancers, potentially assisting with diagnosis and providing early genetic information to inform individualized lung cancer treatments. These approaches may help us enhance treatment outcomes, while reducing the negative effects of systemic chemotherapy.

## Disclosure

The authors report no conflict of interest.
